# Importance of extensive staging in patients with mucosa-associated lymphoid tissue (MALT)-type lymphoma

**DOI:** 10.1054/bjoc.2000.1308

**Published:** 2000-07-24

**Authors:** M Raderer, F Vorbeck, M Formanek, C Österreicher, J Valencak, M Penz, G Kornek, G Hamilton, B Dragosics, A Chott

**Affiliations:** Departments of Internal Medicine I, Division of Oncology, Radiology, Otolaryngology, Internal Medicine IV, Clinical Pathology, University of Vienna, Waehringer Guertel 18–20 A-1090, Vienna; Ludwig Boltzmann Institute for Clinical Oncology, KH Lainz, Vienna, Austria

**Keywords:** MALT-type lymphoma, multifocal involvement, staging

## Abstract

Lymphoma of the mucosa-associated lymphoid tissue (MALT) type usually arises in MALT acquired through chronic antigenic stimulation triggered by persistent infection and/or autoimmune processes. Due to specific ligand–receptor interactions between lymphoid cells and high-endothelial venules of MALT, both normal and neoplastic lymphoid cells display a pronounced homing tendency to MALT throughout the body. In the case of neoplastic disease these homing properties may be responsible for lymphoma dissemination among various MALT-sites. According to this concept, we have standardized staging procedures in all patients diagnosed with MALT-type lymphoma. All patients with MALT-type lymphoma underwent standardized staging procedures before treatment. Staging included ophthalmologic examination, otolaryngologic investigation, gastroscopy with multiple biopsies, endosonography of the upper gastrointestinal tract, enteroclysis, colonoscopy, computed tomography of thorax and abdomen and bone marrow biopsy. Biopsy was performed in all lesions suggestive for lymphomatous involvement, and evaluation of all biopsy specimens was performed by a reference pathologist. 35 consecutive patients with histologically verified MALT-type lymphoma were admitted to our department. Twenty-four patients (68%) had primary involvement of the stomach, five (15%) had lymphoma of the ocular adnexa, three (8.5%) had lymphoma of the parotid, and three (8,5%) of the lung. Lymph-node involvement corresponding to stage EII disease was found in 13 patients (37%), only one patient with primary gastric lymphoma had local and supradiaphragmatic lymph-node involvement (stage EIII). Bone marrow biopsies were negative in all patients. Overall, eight of 35 patients (23%) had simultaneous biopsy-proven involvement of two MALT-sites: one patient each had lymphoma of parotid and lacrimal gland, conjunctiva and hypopharynx, conjunctiva and skin, lacrimal gland and lung, stomach and colon, and stomach and lung. The remaining two patients had bilateral parotideal lymphoma. Staging work-up was negative for lymph-node involvement in all of these eight patients. The importance of extensive staging in MALT-type lymphoma is emphasized by the demonstration of multiorgan involvement in almost a quarter of patients. In addition, our data suggest that extra-gastrointestinal MALT-type lymphoma more frequently occurs simultaneously at different anatomic sites than MALT-type lymphoma involving the GI-tract. © 2000 Cancer Research Campaign


*British Journal of Cancer* (2000) **83**(4), 454–457
© 2000 Cancer Research Campaign
doi: 10.1054/ bjoc.2000.1308, available online at http://www.idealibrary.com on IDEAL®

### Importance of extensive staging in patients with mucosa-associated lymphoid tissue (MALT)-type lymphoma

**M Raderer[1], F Vorbeck[2], M Formanek[3], C Österreicher[4], J Valencak[5], M Penz[1], G Kornek[1], G Hamilton[6], B Dragosics[4] and A Chott[5]**

Departments of Internal Medicine I, [1]Division of Oncology, [2]Radiology, [3]Otolaryngology, [4]Internal Medicine IV, [5]Clinical Pathology, University of Vienna Waehringer Guertel 18–20 A-1090 Vienna; [6]Ludwig Boltzmann Institute for Clinical Oncology, KH Lainz, Vienna, Austria

Lymphoma of the mucosa-associated lymphoid tissue (MALT) type usually arises in MALT acquired through chronic antigenic stimulation triggered by persistent infection and/or autoimmune processes. Due to specific ligand–receptor interactions between lymphoid cells and high-endothelial venules of MALT, both normal and neoplastic lymphoid cells display a pronounced homing tendency to MALT throughout the body. In the case of neoplastic disease these homing properties may be responsible for lymphoma dissemination among various MALT-sites. According to this concept, we have standardized staging procedures in all patients diagnosed with MALT-type lymphoma. All patients with MALT-type lymphoma underwent standardized staging procedures before treatment. Staging included ophthalmologic examination, otolaryngologic investigation, gastroscopy with multiple biopsies, endosonography of the upper gastrointestinal tract, enteroclysis, colonoscopy, computed tomography of thorax and abdomen and bone marrow biopsy. Biopsy was performed in all lesions suggestive for lymphomatous involvement, and evaluation of all biopsy specimens was performed by a reference pathologist. 35 consecutive patients with histologically verified MALT-type lymphoma were admitted to our department. Twenty-four patients (68%) had primary involvement of the stomach, five (15%) had lymphoma of the ocular adnexa, three (8.5%) had lymphoma of the parotid, and three (8,5%) of the lung. Lymph-node involvement corresponding to stage EII disease was found in 13 patients (37%), only one patient with primary gastric lymphoma had local and supradiaphragmatic lymph-node involvement (stage EIII). Bone marrow biopsies were negative in all patients. Overall, eight of 35 patients (23%) had simultaneous biopsy-proven involvement of two MALT-sites: one patient each had lymphoma of parotid and lacrimal gland, conjunctiva and hypopharynx, conjunctiva and skin, lacrimal gland and lung, stomach and colon, and stomach and lung. The remaining two patients had bilateral parotideal lymphoma. Staging work-up was negative for lymph-node involvement in all of these eight patients. The importance of extensive staging in MALT-type lymphoma is emphasized by the demonstration of multiorgan involvement in almost a quarter of patients. In addition, our data suggest that extra-gastrointestinal MALT-type lymphoma more frequently occurs simultaneously at different anatomic sites than MALT-type lymphoma involving the GI-tract. © 2000 Cancer Research Campaign

**Keywords**: MALT-type lymphoma; multifocal involvement; staging

Lymphoma of the mucosa-associated lymphoid tissue (MALT), as introduced by Isaacson and Wright in the early 1980s, is a distinct clinicopathologic entity with characteristic histologic features (Isaacson and Wright, 1983). The MALT lymphoma concept suggests that these tumours, which may be of low- or high-grade malignancy, correspond to cells of postfollicular differentiation stages (Qin et al, 1995) and usually arise in the background of chronic antigenic stimulation triggered by persistent infections and/or autoimmune processes (Greiner et al, 1994). The majority of MALT-type lymphomas occur in the stomach, but this type of lymphoma may affect virtually every organ in the human body, including the intestine, salivary glands, thyroid, lung and ocular adnexa, but also, though less frequently, the skin, urinary bladder and the gonads (Zucca et al, 1997; Isaacson and Norton, 1994*a*). MALT-type lymphomas usually exhibit a tendency to remain localized within the affected MALT-organ for a prolonged period of time, resulting in a favourable prognosis as compared to nodal lymphomas (Thieblemont et al, 1997).

The sites at which MALT-type lymphomas arise are usually devoid of lymphoid tissue, however, chronic antigenic stimulation may induce the accumulation of MALT which prepares the background for the development of MALT-type lymphoma. Activated normal mucosal lymphocytes preferentially recirculate to the mucosa (‘homing’) by binding of their surface receptor $\alpha 4\beta 7$ to the respective ligand on mucosal venule endothelium (Berlin et al, 1993). Similar traffic-control mechanisms appear to be operative also in MALT-type lymphoma, as recently demonstrated in a case of primary gastric lymphoma with secondary intestinal spread (Isaacson and Norton, 1994*a*; 1994*b*; Isaacson, 1999). Since dissemination within MALT may occur without generating overt clinical manifestations, thorough staging of patients to rule out multifocal involvement before initiation of therapy seems mandatory once MALT-type lymphoma has been diagnosed.

This is especially important in view of the fact that individual forms of treatment such as eradication of *Helicobacter pylori* (HP), radiation or chemotherapy are appropriate in different stages

*Received 19 October 1999*
*Revised 2 March 2000*
*Accepted 13 April 2000*

*Correspondence to:* M Raderer of the disease (Haim et al, 1995; Neubauer et al, 1997; Schechter et al, 1998), and therefore strongly depend on meticulous performance of exact staging. In fact, synchronous MALT-type lymphoma at different sites has repeatedly been reported, as has the propensity for late relapse even after decades in patients undergoing local treatment after initial diagnosis (Kawamata et al, 1995; Grazade et al, 1998; Stephen et al, 1998). As MALT may be acquired in several organs, pre-therapeutic work-up as well as follow-up should be quite extensive. We have therefore standardized staging procedures for patients with MALT-type lymphoma at our department and present the results obtained with this extensive staging routine between January 1997 and January 1999.

### PATIENTS AND METHODS

Between January 1997 and January 1999, 35 patients with a histologically verified diagnosis of MALT-type lymphoma were admitted to our department. Upon admission, all biopsy specimens obtained were evaluated by a reference pathologist to verify the presence of MALT-type lymphoma before initiation of staging. Histologic diagnosis of low-grade lymphoma of MALT-type was performed according to the criteria outlined by Isaacson (Isaacson 1983; Isaacson and Norton, 1994*a*). In addition, immunologic phenotyping on paraffin sections was done for demonstration of light-chain restriction and the phenotype CD20+CD5–CD10-cyclinD1-which, in context with the microscopic appearance, is consistent with low-grade B-cell lymphoma of MALT-type. The diagnosis of high-grade lymphoma was based on the presence of large cells with blastic appearance growing in sheets often between glands, with or without a low-grade component, and was also confirmed by phenotyping. In patients with a predominance of a large-cell component, the presence of a low-grade component defined the MALT-origin of the lymphoma, and also pure large-cell lymphomas (diffuse large-cell lymphomas) without evidence of extragastric spread beyond contiguous lymph nodes were included. While the presence of primary diffuse large-cell lymphoma without a low-grade background is not unequivocally felt to be pathogenetically related to MALT-type lymphoma (Chan et al, 1998), cytological and clinical features of such lymphomas arising within a low-grade background are similar to those lesions without a low-grade component. Thus, for the time being, it appears reasonable to classify the latter as primary high-grade MALT-type lymphomas (Isaacson and Norton, 1994*a*; 1994*b*; Isaacson, 1999), and these patients were also included in our series.

All patients diagnosed with MALT-type lymphoma underwent extensive staging before initiation of therapy. Staging consisted of ophthalmologic examination, otolaryngologic investigation including sonography of the salivary glands or magnetic resonance imaging if indicated, gastroscopy with multiple biopsies, endosonography of the upper GI-tract, enteroclysis, colonoscopy, and bone marrow biopsy. CT-scans were evaluated by a reference radiologist, and endoscopies and endosonography were performed by a single individual, as was otolaryngologic examination to rule out inter-observer variabilities. In case of lesions suggestive for lymphomatous involvement, biopsy was attempted in all cases, and all biopsy specimens were again evaluated by a reference pathologist. Staging was performed according to the Ann Arbor staging system modified by Mushoff (1977).

### RESULTS

Among this series of 35 patients (Figure 1), the stomach was the initial site of disease upon diagnosis in 24 patients (68%). Eleven of these patients each had low-grade MALT-type lymphoma or high-grade lymphoma, while two had high-grade disease arising within a low-grade component. Eleven of the 35 patients (32%) had primary extragastric MALT-type lymphoma, occurring in the ocular adnexa ($n$ = 5), the parotid gland ($n$ = 3) and the lung ($n$ = 3).

Staging work-up revealed nodal involvement in 14 of the 35 patients (43%), 13 had stage EII disease and one patient with gastric lymphoma had enlarged lymph nodes on both sides of the diaphragm (stage EIII). In 12 of the patients with stage EII disease, lymph-node enlargement was identified both by CT scan and endosonography, and afflicted lymph nodes were identified by endosonography in one patient. In the patient with stage EIII disease, spiral CT-scan showed enlarged lymph nodes on both sides of the diaphragm, while conventional chest X-ray was not suggestive of enlarged lymph nodes.

In total, eight patients of 35 (23%) had involvement of at least two MALT-organs as documented by staging and consecutive histology (Figure 1). Two patients with primary low-grade gastric MALT-type lymphoma were found to have multifocal disease, involving colon and lung, respectively. Histologic examination of the biopsy samples obtained during colonoscopy and fine-needle biopsy of the lung showed low-grade MALT-type lymphoma corresponding to the histology seen in the gastric lesion. No patient with primary high-grade gastric lymphoma had evidence of multiorgan involvement.

A relatively high proportion of multifocal disease, however, was found in patients with extragastric primaries. In four patients presenting with initial MALT-type lymphoma of the ocular adnexa (located in the conjunctiva and the lacrimal gland in two patients each), synchronous involvement of skin, hypopharynx, parotid and lung was discovered. Again, biopsies obtained from these lesions disclosed low-grade MALT-type lymphoma. In the patient with involvement of both conjunctiva and hypopharynx, the presence of a CD5+ MALT-type lymphoma was disclosed in both locations. In addition, two patients were found to have bilateral MALT-type lymphoma of the parotid as diagnosed by MRI after superficial unilateral parotidectomy in both cases, and was subsequently verified by contralateral biopsy.

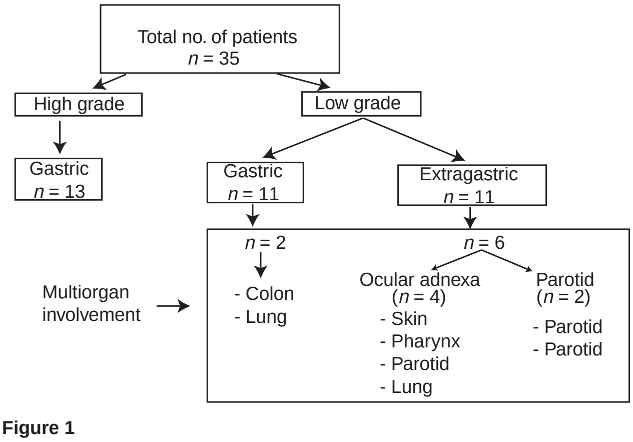

**Figure 1**

### DISCUSSION

MALT-type lymphoma is a relatively rare disease, but nevertheless represents the third most common lymphoma type, accounting for about 7% of all non-Hodgkin's lymphomas (Pileri et al, 1998). Recent years have seen major advances in uncovering a causative role of HP in gastric MALT-type lymphoma along with the potential for complete disappearance of lymphoma after successful eradication of HP in patients with low-grade disease (Isaacson, 1999; Roggerc et al, 1995). According to the recent literature, however, benefit from HP-eradication as sole management seems to be restricted to patients with early-stage disease confined to mucosa and submucosa. In addition, promising results have been reported with application of local radiotherapy in patients with localized lymphoma (Schechter et al, 1998), which would nevertheless result in undertreatment of patients with disseminated disease.

The propensity for mucosal generalization of MALT-type lymphoma has repeatedly been extrapolated from reports demonstrating distant mucosal relapse years after successful local treatment for MALT-type lymphoma (Stephen et al, 1998; Kawamata et al, 1995). In addition, pathologic investigations have clearly shown multifocal involvement of the gastric mucosa in all gastrectomy specimens investigated by PCR (Wotherspoon et al, 1992; Sackmann et al, 1997; Du et al, 1999) even in the absence of clinically and histologically detectable intragastric dissemination. However, the exact percentage of patients affected by clinically apparent multiorgan disease upon diagnosis is not known in detail, as is the risk of relapse within MALT after treatment. Our series allows a rough estimation of the risk for multiorgan involvement in patients diagnosed with MALT-type lymphoma and clearly underscores the importance of extensive staging. Overall, multiorgan involvement was found in almost a quarter of patients (eight of 35) admitted at our institution in a 2-year interval. None of these eight patients, however, had lymph-node involvement, which was detected in a total of 14 patients (43%).

Interestingly only two of 24 patients with gastric MALT-type lymphoma were found to have multifocal disease, whereas six of 11 patients (55%) with extragastric primary manifestations were identified to have synchronous involvement of different anatomic sites, including two patients with bilateral parotid lymphoma. While the occurrence of bilateral parotid lymphoma has repeatedly been described and might be rated as multifocal involvement within the same organ as also seen in gastric MALT-type lymphoma, we could also demonstrate involvement of both the parotid and the contralateral lacrimal gland in one patient.

In addition, no case of multiorgan disease could be found among high-grade lymphoma patients, again raising the question about pathogenetic differences between these lymphomas and MALT-lymphomas without a high-grade component.

Our data suggest a relatively high rate of multiorgan involvement in non-gastric MALT-type lymphomas as opposed to MALT-lymphoma involving the gastrointestinal tract. However, one might argue that these data are the result of a diagnostic bias due to easier accessibility of extra-intestinal MALT-organs such as ocular adnexa or parotid as compared to the GI-tract, resulting in a different latency to clinical manifestation and diagnosis. Recent findings nevertheless indicate putative regionalization in circulation of lymphoid cells between different MALT-sites (Brandtzaeg et al, 1999), providing the cellular and molecular basis for our clinical findings. In fact, lymphoid cells derived from extra-intestinal MALT prefer to circulate through mucosae outside the GI-tract, whereas the opposite applies to GI-MALT, where lymphoid cells possess a low affinity for non-GI MALT (Brandtzaeg et al, 1999).

**Table 1** Staging routine for patients with MALT-type lymphoma

| |
|---|
| Imaging of salivary glands and lacrimal glands (by MRI and/or ultrasound) |
| Ear, nose and throat investigation |
| Gastroscopy with mapping biopsies |
| Endosonography of the upper GI-tract |
| Double-contrast X-ray of the small bowel |
| Colonoscopy |
| Computed tomography of thorax and abdomen |
| Bone marrow biopsy |

Taken together, a standardized staging procedure as performed in our series (Table 1) seems mandatory before initiation of therapy in patients with MALT-type lymphoma in general and in patients with extra-gastrointestinal MALT lymphoma in particular. Demonstration of multiorgan involvement appears to be an important clinical information before initiation of therapy, since such patients are unlikely to benefit from local therapy such as irradiation in extra-gastric sites, and to the current knowledge are not suitable candidates for eradication of HP as sole treatment modality in case of gastric origin of the lymphoma.

### REFERENCES

Berlin C, Berg E, Briskin M, et al (1993) α4β7 intergrin mediates lymphocyte binding to the mucosal vascular addressin MAdCAM-1. *Cell* **74**: 185–195

Brandtzaeg P, Farstad I and Haraldsen G (1999) Regional specialization of the mucosal immune system: primed cells do not always home along the same track. *Immunol Today* **20**: 267–277

Chan W, Wong N, Chan A, et al (1998) Consistent copy number gain in chromosome 12 in primary diffuse large cell lymphomas of the stomach. *Am J Pathol* **152**: 11–16

Du M, Diss TC, Dogan A, et al (1999) Clone specific PCR reveals wide dissemination of gastric MALT lymphoma to the gastrointestinal mucosa. *Mod Pathol* **12**: 135A (abst)

Graziadei G, Pruneri G, Carboni N, et al (1998) Low-grade MALT-lymphoma involving multiple mucosal sites and bone marrow. *Ann Hematol* **76**: 81–83

Greiner A, Marx A, Heesemann J, et al (1994) Idiotype idendity in a MALT-type lyphoma and B-cells in Helcobacter-pylori-associated chronic gastritis. *Lab Invest* **70**: 572–578

Haim N, Leviov M, Ben-Arieh Y, et al (1995) Intermediate and high-grade gastric non-Hodgkin's lymphoma: a prospective study of non-surgical treatment with primary chemotherapy, with or without radiotherapy. *Leuk Lymphoma* **17**: 321–326

Isaacson PG (1999) Gastric MALT lymphoma: from concept to cure. *Ann Oncol* **10**: 637–645

Isaacson PG and Wright DH (1983) Malignant lymphoma of mucosa-associated lymphoid tissue. A distinctive type of B-cell lymphoma. *Cancer* 1983; **52**: 1410–1416

Isaacson PG, Norton AJ (1994*a*) Mucosa-associated lymphoid tissue (MALT) and the MALT-lymphoma concept. In: *Extranodal Lymphomas*. Isaacson PG, Norton AJ (eds), pp. 5–14. Churchill Livingstone: Edinburgh pp. 5–14

Isaacson PG, Norton AJ (1994*b*) Malignant lymphoma of the gastrointestinal tract. In: *Extranodal Lymphomas*. Isaascson PG, Norton AJ (eds), pp. 15–65. Churchill Livingstone: Edinburgh

Kawamata N, Miki T, Fukuda T, et al 1995 Determination of a common clonal origin of gastric and pulmonary mucosa-associated lymphoid tissue lymphomas presenting five years apart. *Intern Med* **34**: 220–223

Musshoff K 1977 Klinische Stadieneinteilung der Non-Hodgkin Lymphome. *Strahlentherapie* **153**: 218–221

Neubauer A, Thiede C, Morgner A, et al 1997 Cure of Helicobacter pylori infection and duration of remission of low-grade gastric mucosa-associated lymphoid tissue lymphoma. *J Natl Cancer Inst* **89**: 1350–1355

 © 2000 Cancer Research Campaign

Pileri S, Milani M, Fraternali-Orcioni G, et al 1998 From the REAL-classificatio to the upcoming WHO-scheme: A step towards universal categorization of lymphoma entities? *Ann Oncol* **9**: 607–612
Qin Y, Greiner A, Trunk MJF, et al 1995 Somatic hypermutation in low-grade mucosa-associated lymphoid tissue-type-B-cell lymphoma. *Blood* **86**: 3528–3533
Roggero E, Zucca E, Pinotti G, et al 1995 Eradication of Helicobacter pylori infection in primary low-grade gastric lymphoma of the mucosa-associated lymphoid tissue. *Ann Intern Med* **122**: 767–769
Sackmann M, Morgner A, Rudolph B, et al 1997 Regression of gastric MALT lymphoma after eradication of *Helicobacter pylori* is predicted by endosonographic staging. MALT Lymphoma Study Group. *Gastroenterology* **113**: 1087–1090
Schechter NR, Portlock Cs, Yahalom J. Treatment of mucosa associated lymphoid tissue lymphoma of the stomach with radiation alone. *J Clin Oncol* **16**: 1916–1921
Stephen MR, Farquharson MA, Sharp RA, et al 1998 Sequential MALT lymphomas of the stomach, small intestine and gall bladder. *J Clin Pathol* **51**: 77–79
Thieblemont C, Bastion Y, Berger F, et al 1997 Mucosa-associated lymphoid tissue gastrointestinal and nongastrointestinal lymphoma behavior: Analysis of 108 patients. *J Clin Oncol* **15**: 1624–1630
Wotherspoon A, Doglioni C and Isaacson PG 1992 Low-grade gastric B-cell lymphoma of mucosa associated lymphoid tissue (MALT): a multifocal disease. *Histopathology* **20**: 29–34
Zucca E, Roggera E, Bertoni F, et al 1997 Primary extranodal non-Hodgkin's lymphomas. Part 1: Gastrointestinal, cutaneous and genitourinary lymphomas. *Ann Oncol* **8**: 727–737

*© 2000 Cancer Research Campaign*
